# Supplementary material for: Infection-generated electric field in gut epithelium drives bidirectional migration of macrophages
Source: PLoS Biol. 2019 Apr 9;17(4):e3000044. doi: 10.1371/journal.pbio.3000044 (PMC6456179; doi:10.1371/journal.pbio.3000044)
Supplement: S1 Table — (DOCX) [file pbio.3000044.s001.docx]

**S1 Table.**

| Lectin | Description | Sugar specificity | Source |
| --- | --- | --- | --- |
| GNL | Fluorescein labeled Galanthus Nivalis Lectin | α-Mannose | Vectorlabs, FL-1241 |
| Con A | Fluorescein Labeled Concanavalin A | α-Mannose, D-Glucose | Vectorlabs, FL-1001 |
| SNA | Fluorescein labeled Sambucus Nigra Lectin | N-Acetylgalactosamine | Vectorlabs, FL-1301 |
| RCA-1 | Fluorescein labeled Ricinus Communis Agglutinin I | D-Galactose | Vectorlabs, FL-1081 |
| MAL-2 | Biotinylated Maackia Amurensis Lectin II | N-Acetylneuraminic acid (sialic acid) | Vectorlabs, B-1265 |
