# Supplementary material for: Infection-generated electric field in gut epithelium drives bidirectional migration of macrophages
Source: PLoS Biol. 2019 Apr 9;17(4):e3000044. doi: 10.1371/journal.pbio.3000044 (PMC6456179; doi:10.1371/journal.pbio.3000044)
Supplement: S2 Table — (DOCX) [file pbio.3000044.s002.docx]

**S2 Table.**

| Reagent | Description | Source |
| --- | --- | --- |
| Microspheres | FluoSpheres® Carboxylate-Modified Microspheres, 1.0 µm, blue fluorescent (365/415), 2% solids | Invitrogen, F8814 |
| Microspheres | FluoSpheres™ Carboxylate-Modified Microspheres, 1.0 µm, red fluorescent (580/605), 2% solids | Invitrogen, F8821 |
| Neuraminidase | Neuraminidase (Sialidase) from *Vibrio cholerae* | Roche, 11080725001 |
| Anti-*Salmonella* | Polyclonal antibody | Mybiosource, MBS535017 |
| Phalloidin | Alexa Fluor™ 555 Phalloidin | Invitrogen, A34055 |
| Streptavidin | Fluorescein Streptavidin | Vectorlabs, SA-5001 |
| Aqua blue | LIVE/DEAD™ Fixable Aqua Dead Cell Stain Kit, for 405 nm excitation | Invitrogen, L34957 |
