# Supplementary material for: Infection-generated electric field in gut epithelium drives bidirectional migration of macrophages
Source: PLoS Biol. 2019 Apr 9;17(4):e3000044. doi: 10.1371/journal.pbio.3000044 (PMC6456179; doi:10.1371/journal.pbio.3000044)
Supplement: S3 Table — (DOCX) [file pbio.3000044.s003.docx]

**S3 Table. Plasmids and *Salmonella* strains used in this study**

| Plasmid/Strain | Description and/or Relevant genotype | Reference |
| --- | --- | --- |
| Plasmid |  |  |
| pJC43 | pBBR1-MCS2 based expressing a GFP mut3 under *aphA3* promoter. KanR. | [1] |
| pFT/RalFc | pBBR1-MCS4 based low-copy number plasmid expressing FT::RalF(350-374). CmR. |  |
| pGFT/RalFc | In pFT/RalFc expressing GFP mut3 under *aphA3* promoter. CmR. | This work |
| pCP20 | Express yeast FLP recombinase. Temperature sensitive. CmR, AmpR. |  |
| *Salmonella* |  |  |
| D23580 | ST313, multidrug-resistant, isolated from patient's blood in 2004 in Malawi | [2,3] |
| IR715 | ATCC 14028, Nal^R^ derivative | [4] |
| AJB75 | IR715 derivative, *invA*::Tn*phoA* |  |
| ∆*invA* | AJB75, pGFT/RalFc | This work |
| KLL18 | IR715 derivative, glmS::mCherry St::FRT | This work |
| SL1344 | Virulent laboratory strain |  |
| SL1344St | SL1344, glmS∷mCherry | [5] |
| LT2 | *S. typhimurium* LT2 | [6] |

**References**

1. Celli, J., S.P. Salcedo, and J.P. Gorvel, *Brucella coopts the small GTPase Sar1 for intracellular replication.* Proc Natl Acad Sci U S A, 2005. **102**(5): p. 1673-8.

2. Kingsley, R.A., et al., *Epidemic multiple drug resistant Salmonella Typhimurium causing invasive disease in sub-Saharan Africa have a distinct genotype.* Genome Res, 2009. **19**(12): p. 2279-87.

3. Carden, S.E., et al., *Pseudogenization of the Secreted Effector Gene sseI Confers Rapid Systemic Dissemination of S. Typhimurium ST313 within Migratory Dendritic Cells.* Cell Host Microbe, 2017. **21**(2): p. 182-194.

4. Stojiljkovic, I., A.J. Baumler, and F. Heffron, *Ethanolamine utilization in Salmonella typhimurium: nucleotide sequence, protein expression, and mutational analysis of the cchA cchB eutE eutJ eutG eutH gene cluster.* J Bacteriol, 1995. **177**(5): p. 1357-66.

5. Knodler, L.A., et al., *Noncanonical inflammasome activation of caspase-4/caspase-11 mediates epithelial defenses against enteric bacterial pathogens.* Cell Host Microbe, 2014. **16**(2): p. 249-56.

6. McClelland, M., et al., *Complete genome sequence of Salmonella enterica serovar Typhimurium LT2.* Nature, 2001. **413**(6858): p. 852-6.
