# Supplementary figures and images for: Infection-generated electric field in gut epithelium drives bidirectional migration of macrophages
Source: PLoS Biol. 2019 Apr 9;17(4):e3000044. doi: 10.1371/journal.pbio.3000044 (PMC6456179; doi:10.1371/journal.pbio.3000044)

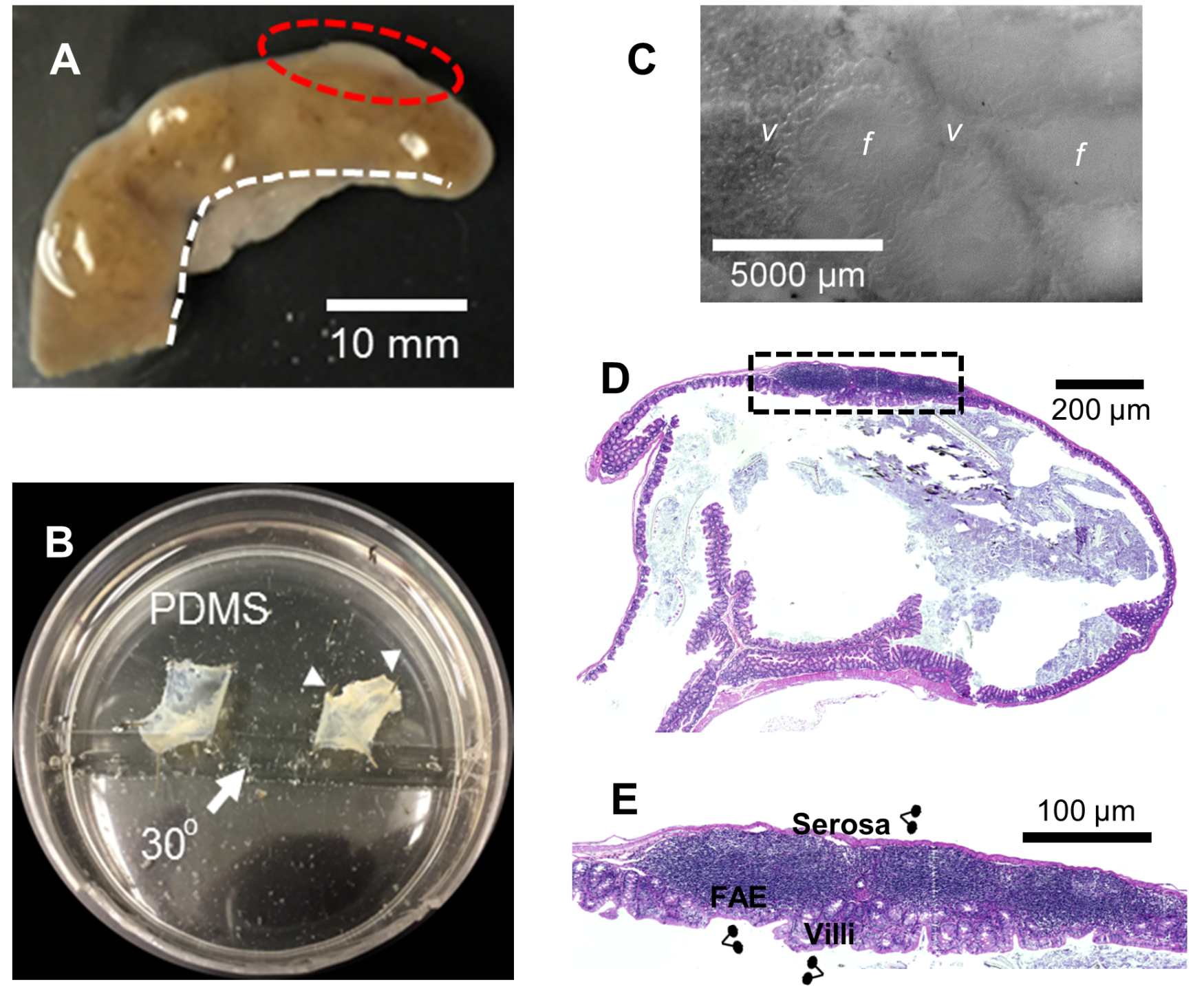

Supplement: S1 Fig — (A) Cecum was dissected from C57BL/6 mouse and opened longitudinally along the mesenteric attachment remnant (dotted white line) to avoid incision damage of lymphatic structure that is located under the antimesenteric mucosa near the apex (red circle). (B) Cecum mounted in a custom-made chamber with mucous epithelium facing up on a 30° slope of silicone gel and held with the cecum edge with fine metal pins (white arrow heads). (C) Mouse cecal epithelium under a dissecting microscope. FAE—the smooth appearing regions (f), and inter-follicle/surrounding villi (v) are shown. Bar, 5,000 μm. (D) HE stain of a mouse cecum showing the structure of a Peyer’s patch. Bar, 200 μm. (E) Magnification of the checked area of panel D showing FAE and inter-follicle and surrounding villi. Double-dotted forks indicate the sites where TEP and JI were measured. Serosal epithelia served as controls. Bar, 100 μm. FAE, follicle-associated epithelium; HE, hematoxylin–eosin; JI, electric current density; TEP, transepithelial potential. (TIF) [file pbio.3000044.s004.tif]

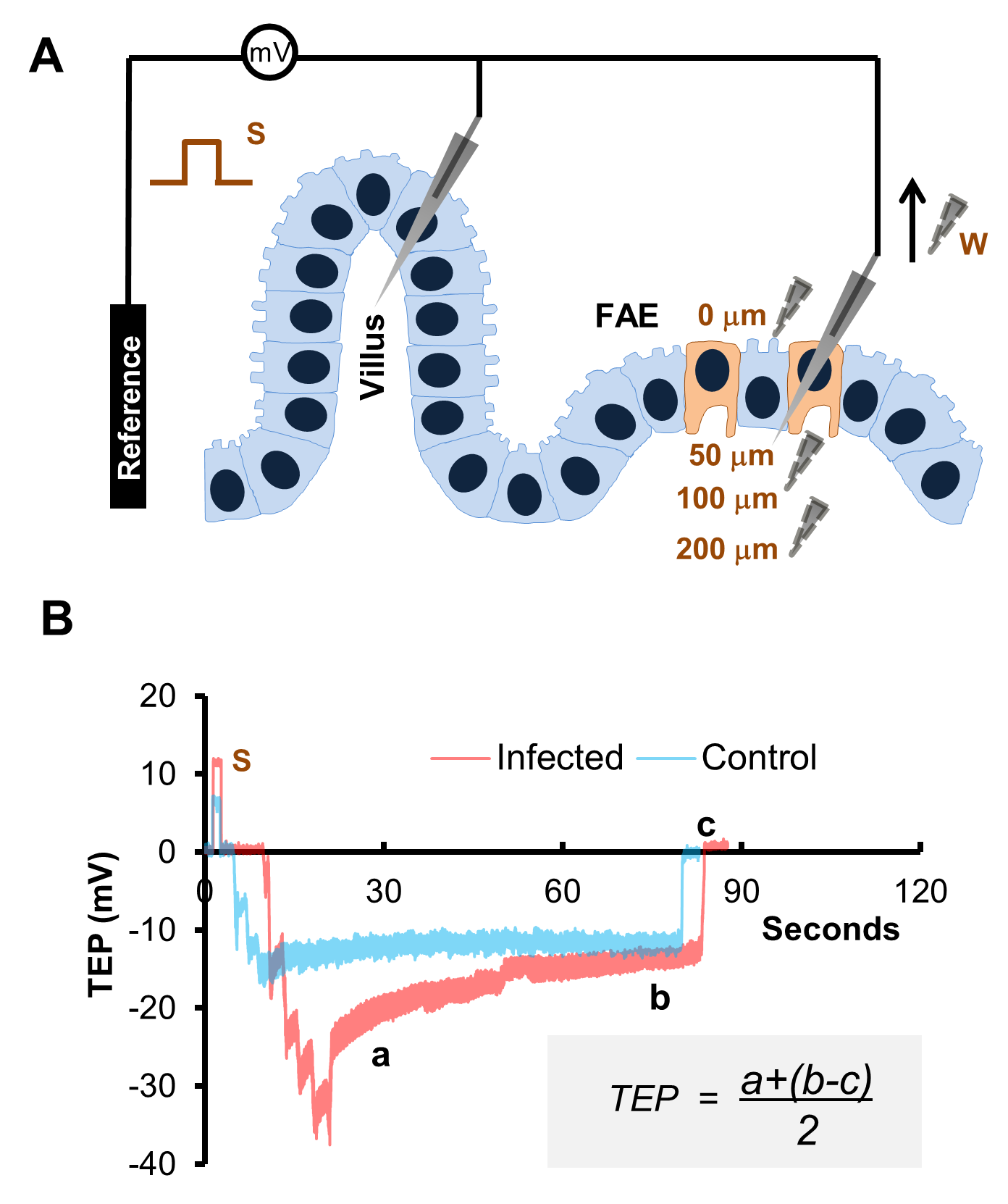

Supplement: S2 Fig — (A) Schematic depicting the microelectrode setup, sites, and procedures of measurement. The measuring microelectrode was impaled through the FAE or surrounding villus epithelial layer (one site at a time), and the circuit was closed by a reference electrode placed in the buffer, representing the lumen. Microelectrode resistance (S, in which 10 mV equals 1 MΩ) was generated and recorded prior to each impalement to ensure that the tip was neither broken nor obstructed. In some cases, the TEP of infected FAEs were measured as follows: first at the epithelial surface (0 μm), then in stepwise at 50, 100, and 200 μm in depth. The potential typically returns to the baseline of 0 mV after microelectrode withdrawal (W). (B) Two representative traces and a specific equation used to calculate TEP value as a modified mean from S1 Data. In the equation, “a” and “b’”are the early and late values of each impalement in which the electrode was kept in position for at least 60 s. “c” is the reference value immediately after the electrode withdrawal. FAE, follicle-associated epithelium; TEP, transepithelial potential. (TIF) [file pbio.3000044.s005.tif]

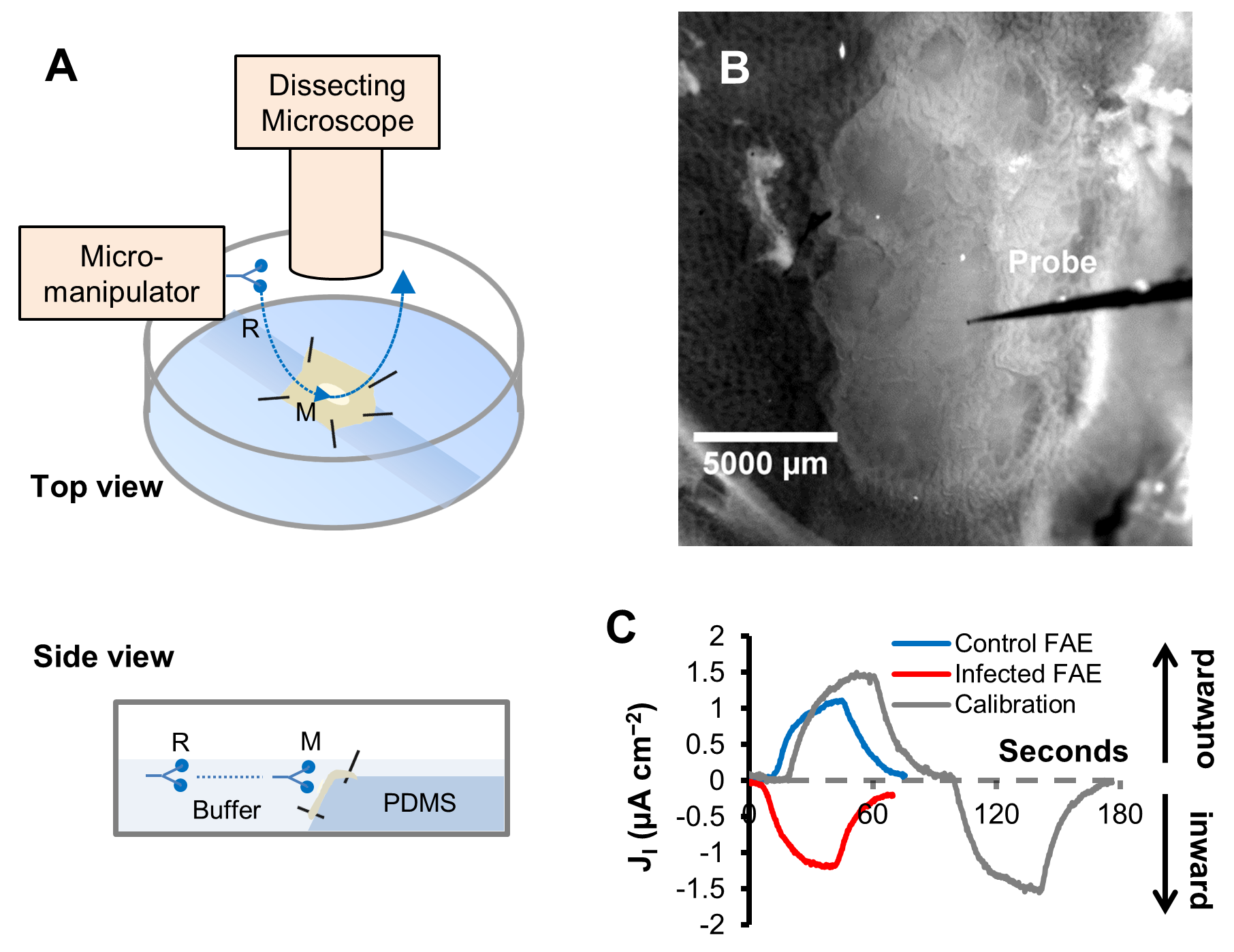

Supplement: S3 Fig — (A) Schematic of equipment setup and measuring procedures. Under a dissecting microscope, a probe vibrating between 100 and 200 Hz controlled by a micromanipulator is moved from the reference position (R) to the measuring position (M), as close as possible (approximately 20 μm) to the epithelial surface, to detect ionic currents. (B) A picture of a vibrating probe approaching an FAE. (C) Representative traces of bioelectric current measured at the FAE from Salmonella-infected or mock-infected control mouse cecum. Probes were calibrated by passing a 1.5 μA cm−2 electric current through the measuring buffer in either direction (see S1 Data). By convention, flux of positive charge is used for electric current direction. As in most studies, we used conventional current flow; therefore outward current density is defined as net positive charge leaving the epithelial surface and inward current densities as that entering. Hence, positive values represent net outward current densities, and negative values represent net inward current densities. FAE, follicle-associated epithelium. (TIF) [file pbio.3000044.s006.tif]

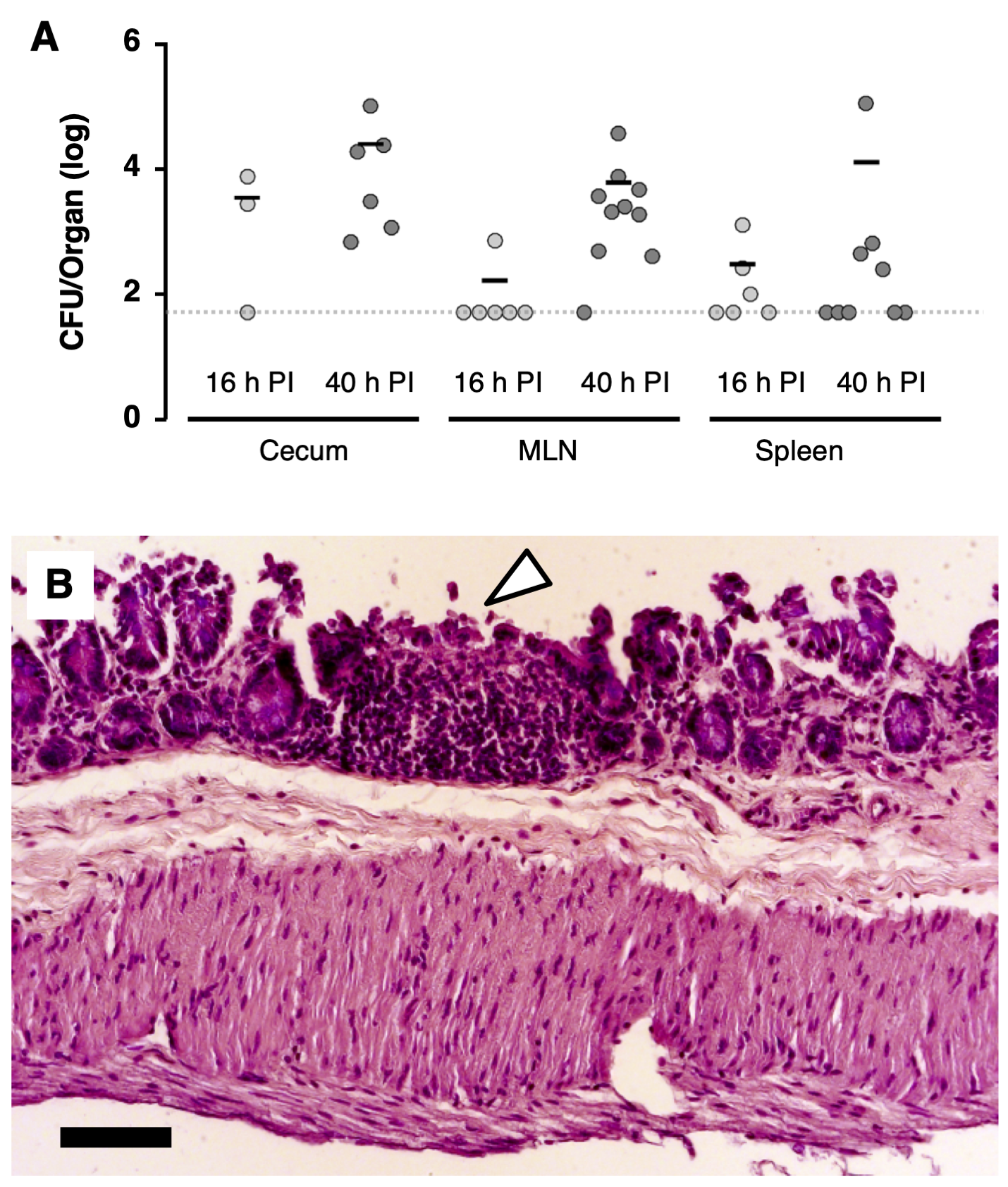

Supplement: S4 Fig — (A) Ceca, free of contents, MLNs and spleens were dissected in sterile conditions at 16 h or 40 h PI from mice orally infected with S. Typhimurium. Bacterial loads were determined by homogenizing each specimen, serial dilution and plating on LB plates. CFUs were calculated by counting bacterial colonies selected with appropriate antibiotics. CFUs lower than the detection limit as indicated by the dotted line were treated as the limit (see S1 Data). (B) HE stain of a cecum section from mice orally infected with S. Typhimurium. Disruption of the FAE (arrowhead) and thickened intestinal wall are shown. Bar, 100 μm. CFU, colony forming unit; FAE, follicle-associated epithelium; HE, hematoxylin–eosin; LB, Luria-Bertani; MLN, mesenteric lymph node; PI, post infection; S. Typhimurium, Salmonella enterica serotype Typhimurium. (TIF) [file pbio.3000044.s007.tif]

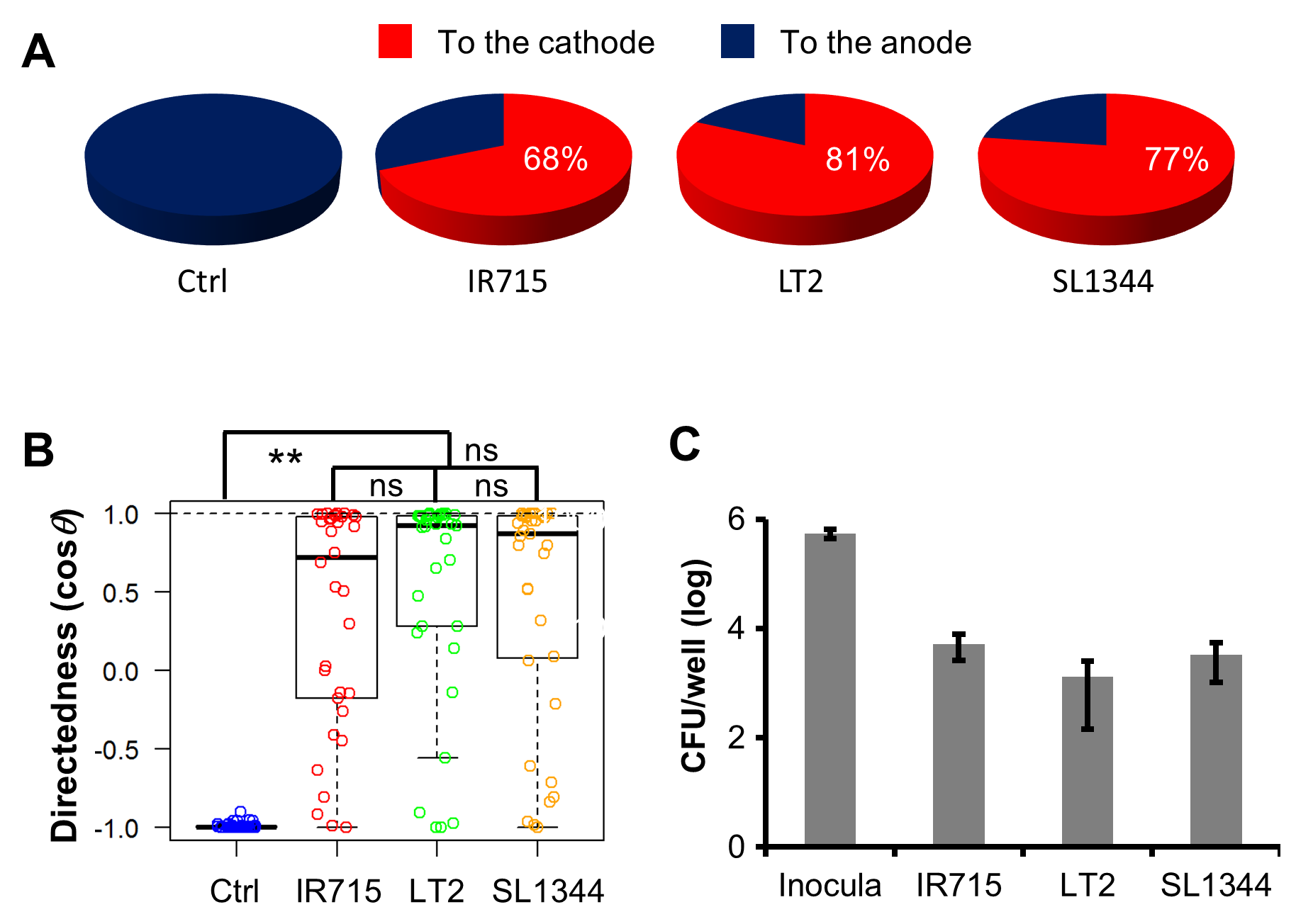

Supplement: S5 Fig — Mouse BMDMs challenged with 3 virulent S. Typhimurium at an MOI of 20, subject to galvanotaxis assay at 16 h PI. EF is 4 V cm−1. Duration is 3 h. (A) Pie charts show percentage of cells migrating to the cathode (red) or to the anode (blue). (B) Overall directionality. **P < 0.01 by one-way ANOVA analysis followed by post hoc Tukey HSD test (see S1 Data). (C) Quantification of intracellular bacteria. BMDMs were seeded in 24-well plates and challenged with S. Typhimurium at an MOI of 20. Actual inocula were determined by plating and colony counting. Intracellular bacteria at 16 h PI was determined by a gentamycin protection assay. Representative data are presented as log CFU per well, normalized to each inoculum. Bar in SE from triplicate wells (see S1 Data). BMDM, bone marrow-derived macrophage; CFU, colony forming unit; EF, electric field; HSD, honest significant difference; MOI, multiplicity of infection; ns, nonsignificant; PI, post infection; S. Typhimurium, Salmonella enterica serotype Typhimurium. (TIF) [file pbio.3000044.s008.tif]

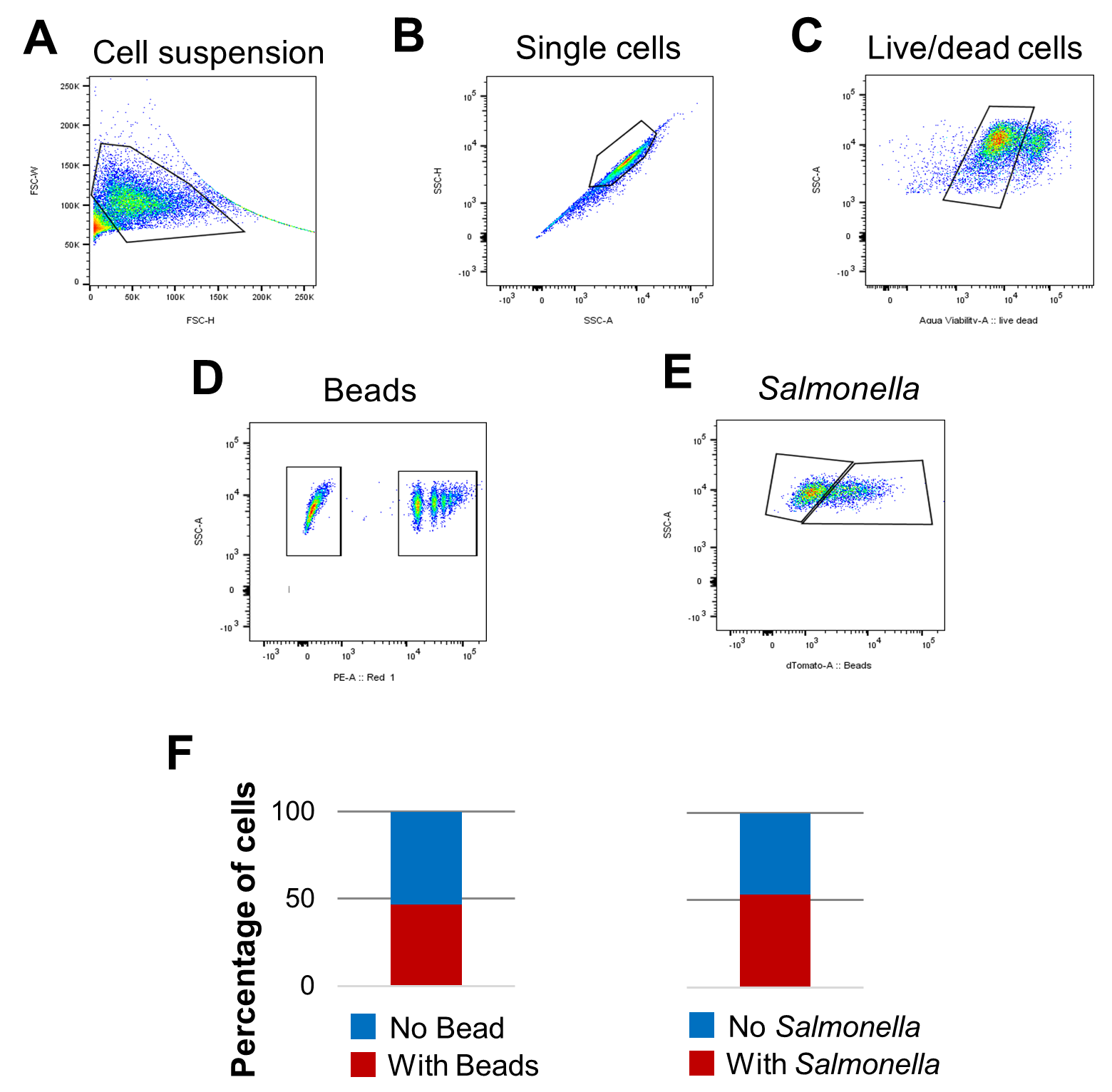

Supplement: S6 Fig — BMDMs were challenged with 1-μm, red fluorescently labeled polystyrene microspheres or live S. Typhimurium IR715 constitutively expressing mCherry at an MOI of 20. Excessive beads and bacteria were removed by washing. Residual extracellular bacteria were killed per gentamycin treatment. (A–E) Representative flow cytograms of a complete experimental design to count target cells using flow cytometry. (A) At 16 h PI, cells were harvested and labeled with Aqua blue and analyzed by flow cytometry. (B) An example of gates used to exclude fragments and cell clumps. (C) Dead cells were excluded by gating Aqua blue signal. Live cells were subject to further cell counting in either PE fluorescence channel for red fluorescent beads (D) or with the dTomato fluorescence channel for Salmonella expressing mCherry (E). (F) Representative bar charts showing percentage of macrophages containing intracellular bacteria or beads. BMDM, bone marrow-derived macrophage; MOI, multiplicity of infection; PE, Phycoerythrin; PI, post infection; S. Typhimurium, Salmonella enterica serotype Typhimurium. (TIF) [file pbio.3000044.s009.tif]

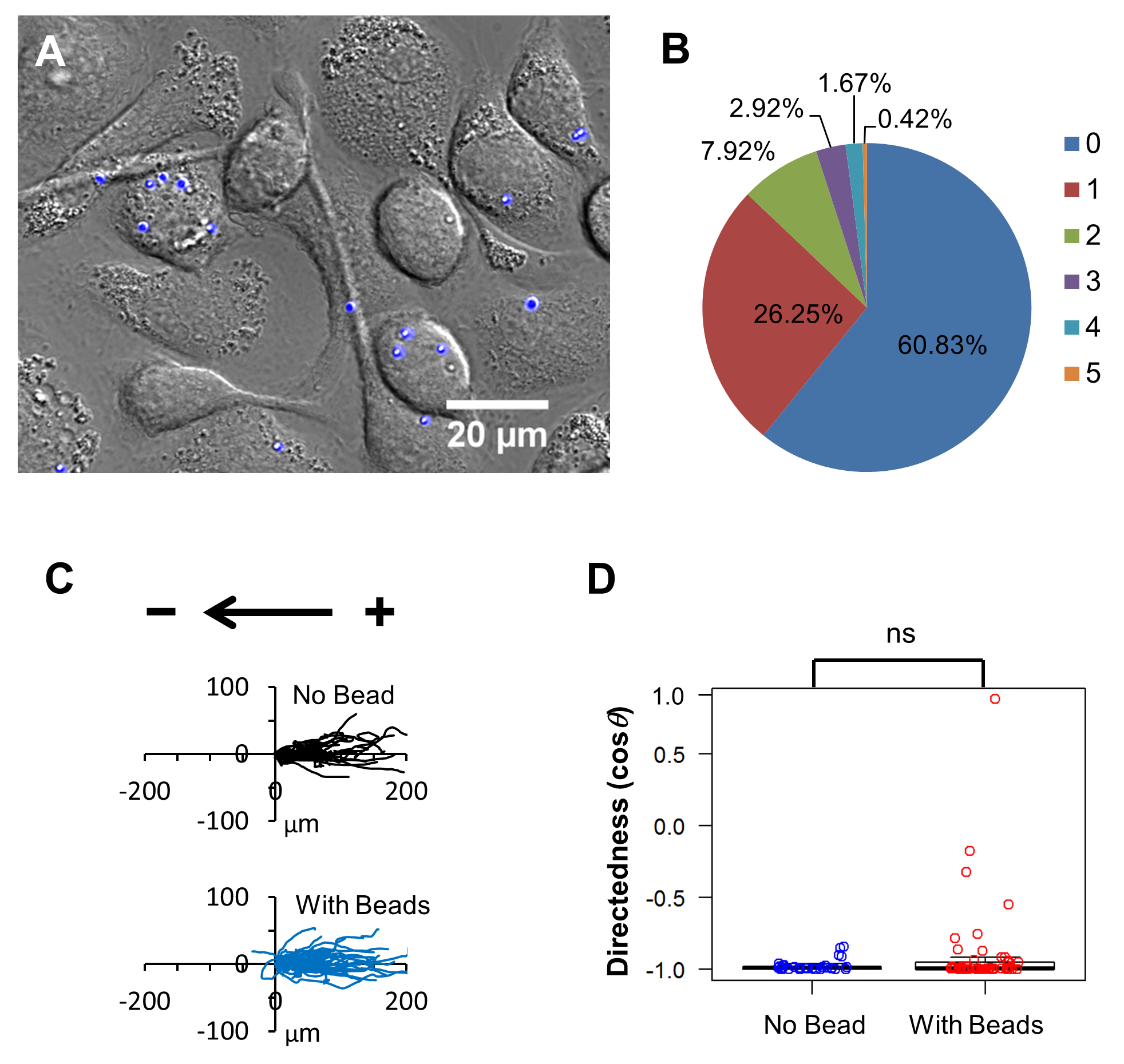

Supplement: S7 Fig — (A) BMDMs were seeded in 96-well glass-bottom plates and challenged with 1-μm microspheres with blue fluorescent at an MOI of 20. Excessive microspheres were removed by washing with medium. At 16 h PI, cells bearing beads (blue) were counted under an epifluorescence microscope. (B) Pie chart of percentage of cells with 0, 1, or up to 5 bead(s) in a typical experiment. (C) Trajectories and (D) directedness of macrophages under an EF of 4 V cm−1 in the indicated orientation for 3 h. ns by unpaired Student t test (see S1 Data). BMDM, bone marrow-derived macrophage; EF, electric field; MOI, multiplicity of infection; ns, nonsignificant; PI, post infection. (TIF) [file pbio.3000044.s010.tif]

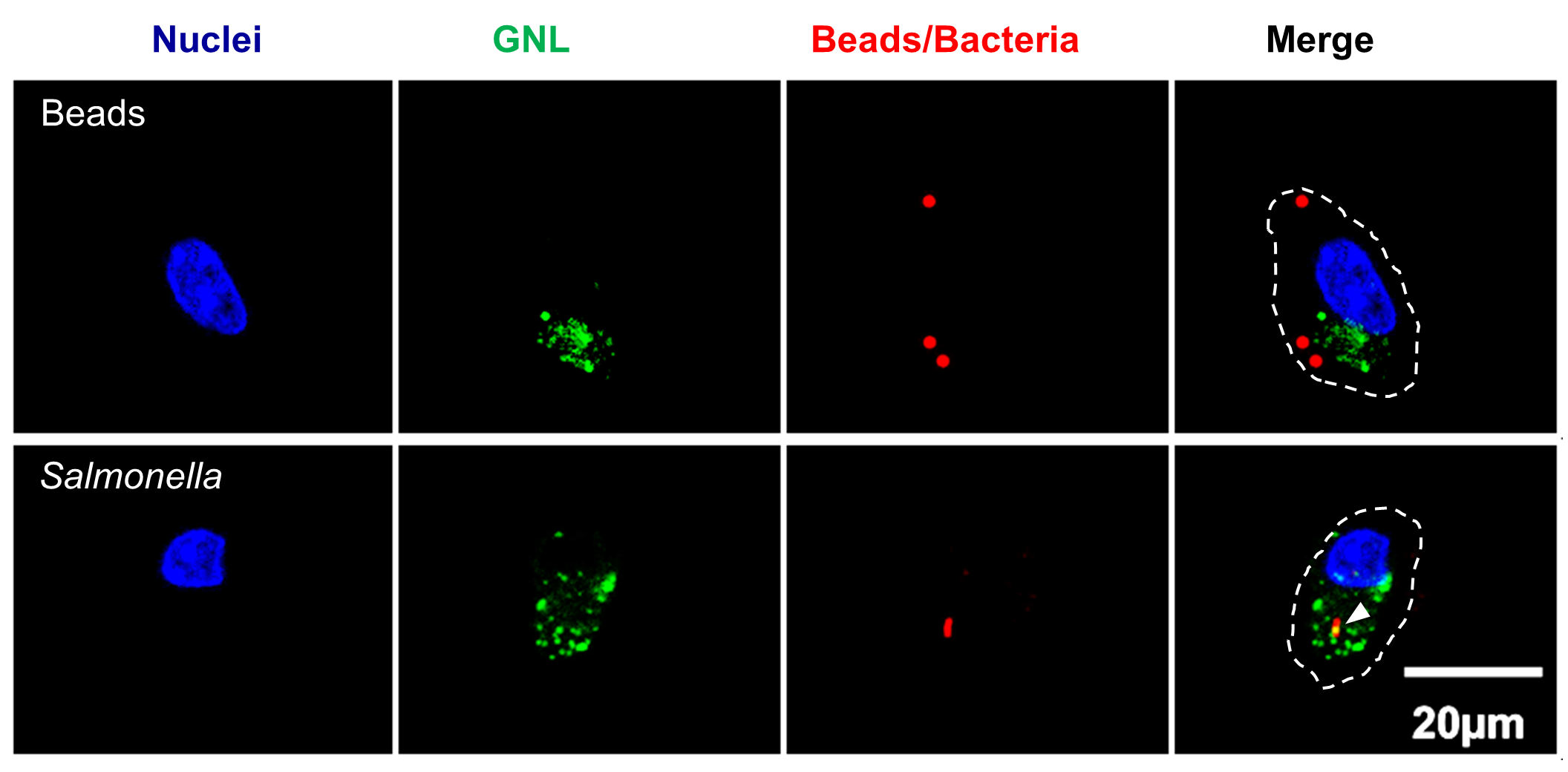

Supplement: S8 Fig — Representative confocal photographs show BMDMs containing red fluorescence-labeled beads or Salmonella expressing mCherry (red). Cells were fixed, permeabilized, and stained with DAPI (blue) and FITC-conjugated GNL (green). Cells were outlined in merged photographs (white dashed line). Bar, 20 μm. Note the GNL-binding aggregates inside macrophages containing intracellular Salmonella (white arrowhead). BMDM, bone marrow-derived macrophage; FITC, fluorescein isothiocyanate; GNL, Galanthus Nivalis lectin. (TIF) [file pbio.3000044.s011.tif]

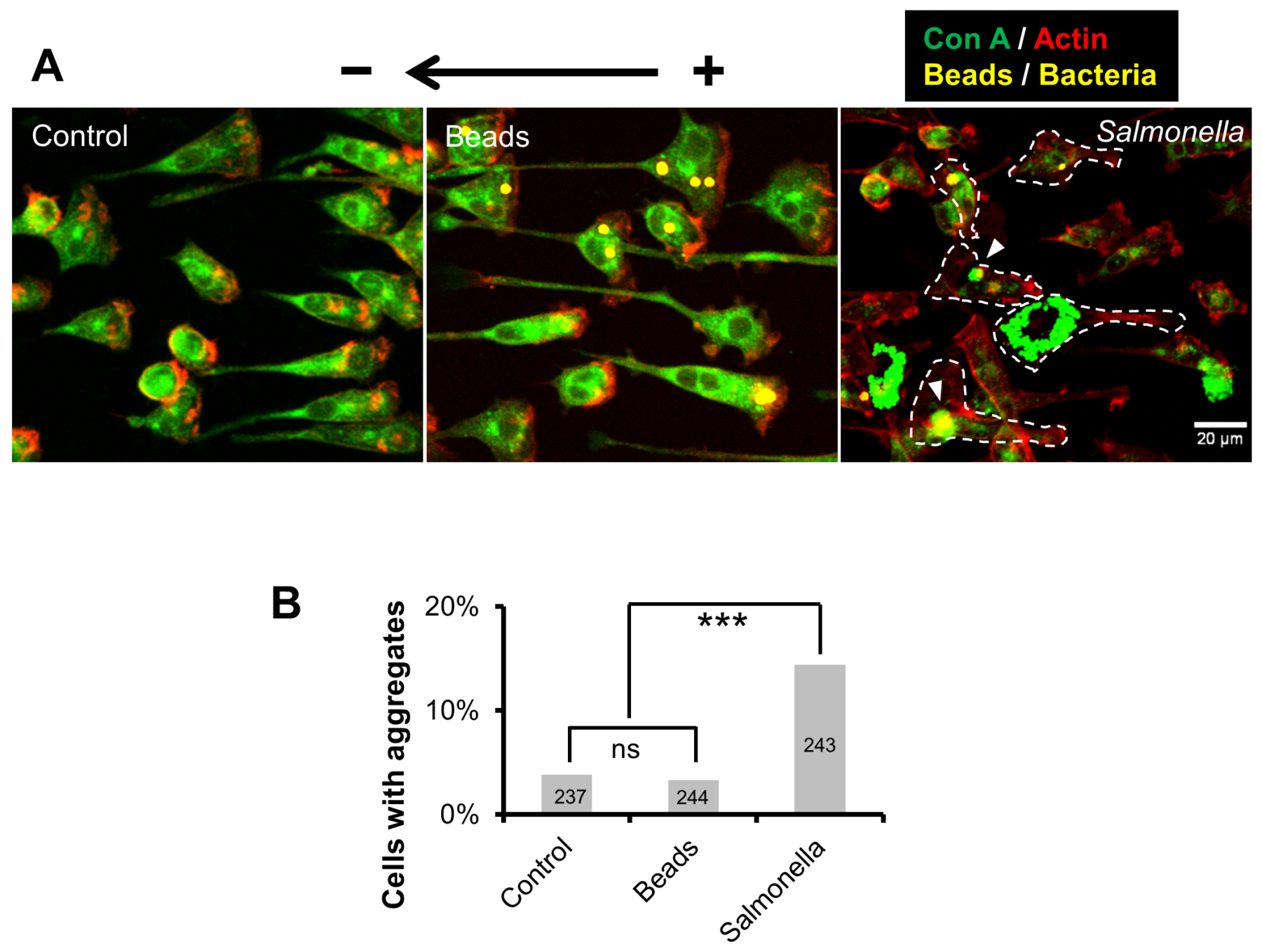

Supplement: S9 Fig — (A) Galvanotaxis assays were performed per the rigorous experiment design illustrated in Fig 3A. BMDMs were fixed, permeabilized, and labeled with Alexa Fluor 555 Phalloidin (red) and FITC-conjugated Con A (green), and scanned with an upright confocal microscope. Phagocytosed beads and intracellular bacteria were pseudocolored in yellow. Bar, 20 μm. Control macrophages (left panel) and macrophages challenged with beads (middle panel) were exclusively polarized to the anode with a characteristic morphology: massive actin meshwork in the front and a uropod at the rear. Cells infected with Salmonella (right panels) reversed their polarity to the cathode. Note the significant Con A–binding aggregates in macrophages containing intracellular Salmonella (white arrowheads). (B) Quantification of macrophages with Con A aggregates. Number of cells counted is indicated inside each bar. ***P < 0.001 by χ2 test (see S1 Data). BMDM, bone marrow-derived macrophage; Con A, concanavalin A; FITC, fluorescein isothiocyanate. (TIF) [file pbio.3000044.s012.tif]

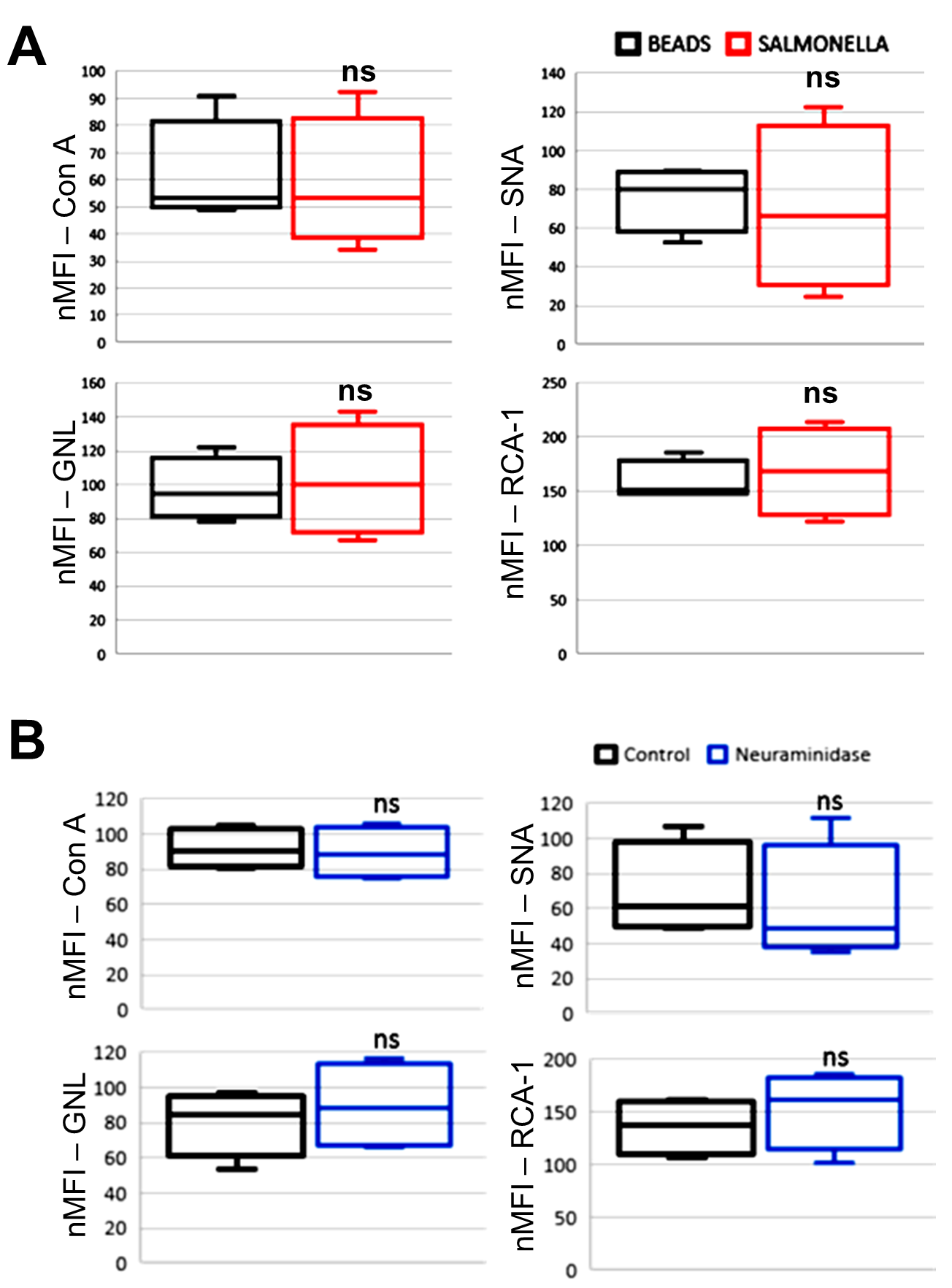

Supplement: S10 Fig — Box plots showing nMFI of macrophages, either (A) challenged with microspheres and Salmonella at 16 h PI or (B) incubated with 0 or 100 mU ml−1 neuraminidase for 30 min. Cells were stained with Con A, SNA, GNL, or RCA-1 and analyzed by flow cytometry. Data from 4 independent experiments. ns by unpaired Student t test (see S1 Data). Con A, concanavalin A; GNL, Galanthus Nivalis lectin; nMFI, normalized mean fluorescence intensity; ns, nonsignificant; PI, post infection; SNA, Sambucus Nigra lectin; RCA-1, Ricinus Communis Agglutinin I. (TIF) [file pbio.3000044.s013.tif]

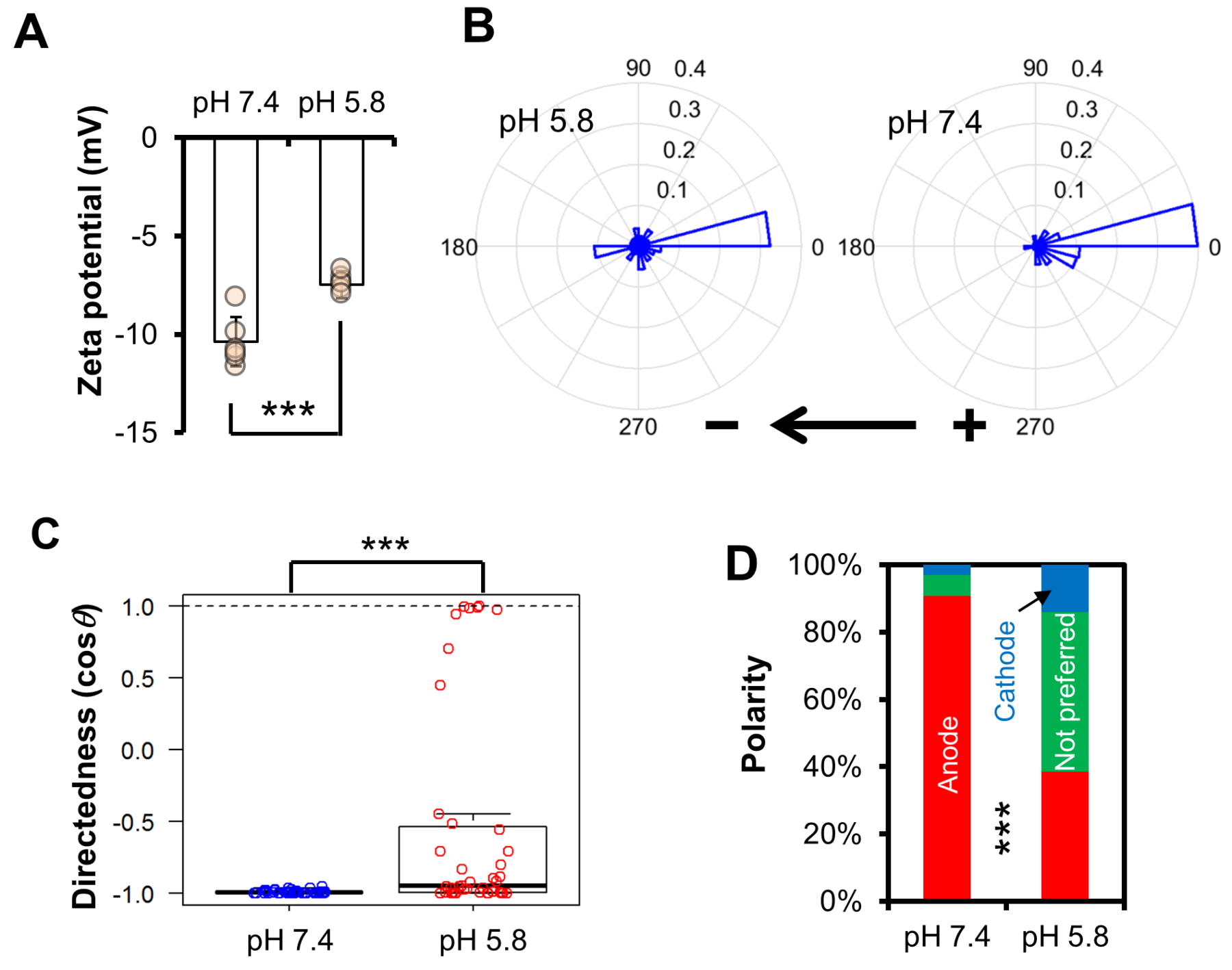

Supplement: S11 Fig — (A) Zeta potential of BMDMs cultured in pH 7.4 or pH 5.8. ***P < 0.001 by Student t test. (B) Rose plots and (C) directedness of BMDMs cultured in pH 7.4 or pH 5.8 exposed to an EF of 4 V cm−1 for 3 h. ***P < 0.001 by Student t test. (D) Polarity of BMDMs cultured in pH 7.4 or pH 5.8 and exposed to an EF of 4 V cm−1 for 3 h. Data were quantified from a representative of 2 independent experiments. ***P < 0.001 by χ2 test (see S1 Data). See also S5 Movie. BMDM, bone marrow-derived macrophage; EF, electric field. (TIF) [file pbio.3000044.s014.tif]
